# Investigation on Filtration Control of Zwitterionic Polymer AADN in High Temperature High Pressure Water-Based Drilling Fluids

**DOI:** 10.3390/gels8120826

**Published:** 2022-12-14

**Authors:** Ren Wang, Jie Yang, Luman Liu, Jianlong Wang, Zhenbo Feng, Die Zhang, Shan Gao, Jiao Wang, Han Ren, Baotong Hui

**Affiliations:** 1 CNPC Engineering Technology R&D Company Ltd., Beijing 102206, China; 2 College of Chemistry and Chemical Engineering, Southwest Petroleum University, Chengdu 610500, China; 3 Daqing Drilling & Exploration Engineering Corporation No. 2 Drilling Co., Daqing 163413, China; 4 Gas Storage Company of Liaohe Oilfield Company, Panjin 124000, China

**Keywords:** water-based drilling fluids, filtration loss reducer, zwitterionic polymer, temperature resistance, salt resistance

## Abstract

With the exploration and development of high-temperature and high-salt deep oil and gas, more rigorous requirements are warranted for the performance of water-based drilling fluids (WBDFs). In this study, acrylamide, 2-acrylamide-2-methylpropanesulfonic acid, diallyl dimethyl ammonium chloride, and N-vinylpyrrolidone were synthesized by free radical copolymerization in an aqueous solution to form a temperature and salt-resistant zwitterionic polymer gel filtration loss reducer (AADN). The zwitterionic polymer had excellent adsorption and hydration groups, which could effectively combine with bentonite through hydrogen bonds and electrostatic attraction, strengthening the hydration film thickness on the surface of bentonite, and promoting the stable dispersion of drilling fluid. In addition, the reverse polyelectrolyte effect of zwitterionic polymers strengthened the drilling fluid’s ability to resist high-temperature and high-salt. The AADN-based drilling fluid showed excellent rheological and filtration control properties (FL_API_ < 8 mL, FL_HTHP_ < 29.6 mL) even after aging at high-temperature (200 °C) and high-salt (20 wt% NaCl) conditions. This study provides a new strategy for simultaneously improving the high-temperature and high-salt tolerance of WBDFs, presenting the potential for application in drilling in high-temperature and high-salt deep formations.

## 1. Introduction

As a critical component in drilling operations, drilling fluid plays a vital role in the entire drilling operation, such as wellbore cleaning, suspending and carrying cuttings, balancing formation pressure, cooling and lubricating drill bits, and protecting oil and gas reservoirs [1,2,3,4]. WBDFs are widely used in drilling engineering because of their low cost, environmental protection, and excellent performance [5,6,7]. With the rapid growth of demand for oil and gas resources and the depletion of oil and gas resources in shallow and medium layers, oil and gas development is gradually developing in deep and ultra-deep layers [8,9,10]. Deep and ultra-deep high temperatures, high pressure, and complex geological conditions (mostly salt-gypsum layer) seriously limit the performance of drilling fluids. Drilling fluid additives are easily degraded, cross-linked, desorbeded, and undergo conformational change under high-temperature and high-salt, resulting in a rheological disorder of the drilling fluid, loss of filtration control, and deterioration of settlement stability [11,12,13,14], thus affecting drilling operations.

As an indispensable additive of WBDFs, a filtration loss reducer can inhibit the aggregation of bentonite particles and maintain reasonable particle size distribution by adsorbing on the surface of bentonite, thus forming a thin and dense mud cake on the sidewall to reduce filtrate loss [2]. Therefore, the filtration loss reducer plays a significant role in controlling the loss of drilling fluids, ensuring wellbore stability, and protecting reservoirs [15,16,17], and has become the current research’s focus. Polymers are the primary filtrate reducers, including natural polymers, modified natural polymers, and synthetic polymers. Natural polymer materials, including xanthan gum, chitosan, modified starch, modified cellulose, and lignin, are widely used as drilling fluid rheology modifiers and filtration loss reducers in shallow and medium layers [18,19,20,21]. However, the molecular structure of glycosidic bonds in natural polymer materials is easily degraded at high temperatures, which limits their application as drilling fluid additives in deep-well drilling.

Synthetic filtration loss reducers such as acrylamide polymers have the characteristics of variable monomer composition and controllable molecular weight, showing excellent high-temperature resistance. However, if the salt components exist simultaneously, they cause polymer chain curl and bentonite aggregation or flocculation, weaken the interaction between polymer and bentonite, and lead to the deterioration of drilling fluid performance [8,22]. Therefore, filtration control of drilling fluids in high-temperature and high-salt environments faces significant technical difficulties. Fortunately, the emergence of zwitterionic polymers has brought a new solution strategy to this challenge. Zwitterionic polymers with anion and cation groups on the molecular chain have high adsorption, hydration ability, and stability [7,23,24] due to the reverse polyelectrolyte effect [25,26]. For example, a zwitterionic polymer (ADD) synthesized with acrylamide, 2-acrylamide-2-methylpropanesulfonic, and diallyl dimethylammonium chloride showed improved rheological and filtration properties at 150 °C and salt conditions [11]. An amphoteric quadripolymer was synthesized using acrylamide, 2-acrylamide-2-methyl propane sulfonic acid, dimethyl diallyl ammonium chloride, and sodium styrene sulfonate, which showed excellent performance at 180 °C [27]. Zwitterionic polymers as filtration loss reducers have progressed in regulating the rheological and filtration properties of drilling fluid under high-temperature and high-salt environments.

**Scheme 1:**
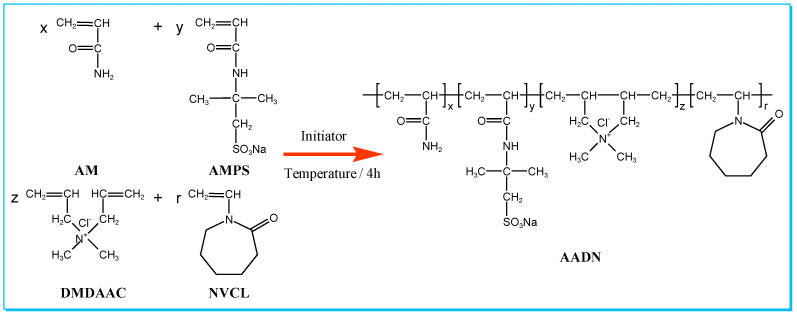
Possible reaction roadmap of AADN.

In this study, a novel zwitterionic polymer gel (AADN) with high-temperature and high-salt tolerance was synthesized by free radical copolymerization of nonionic monomer acrylamide (AM), anionic monomer 2-acrylamide-2-methylpropanesulfonic acid (AMPS), cationic monomer diallyl dimethyl ammonium chloride (DMDAAC) and heterocyclic monomer N-vinylpyrrolidone (NVCL). In the polymer, AM and DMDAAC units as adsorption groups enhanced the adsorption between the polymer and bentonite. As a hydration group, the AMPS unit enhances the hydration and dispersion of bentonite and strengthens the salt tolerance of drilling fluids. The NVCL unit, as the main chain structure, enhances the rigidity and temperature resistance of the polymer molecular chain [28]. A possible reaction mechanism is shown in Scheme 1. The synthesis conditions were optimized by orthogonal experiments, and the zwitterionic polymer showed excellent filtration control efficiency in high-temperature and high-salt (200 °C, 15 wt% NaCl) environments. Our research may provide a new choice for high-temperature and high-salt filtration loss reducers in deep layers.

## 2. Results and Discussion

### 2.1. Optimization of Polymer Synthesis Conditions

According to the principle of free radical polymerization, the factors affecting the synthesis product are monomer molar ratio, monomer concentration, reaction temperature, initiator concentration, system pH, and reaction time. The main influencing factors are monomer molar ratio, reaction temperature, initiator concentration, and total monomer concentration, which were explored through orthogonal experiments. According to our pilot experiment, the pH was determined to be 7, and the reaction time was 4 h. An orthogonal experiment was designed to obtain the optimal reaction conditions for the synthesizing zwitterionic polymer AADN. Furthermore, the synthesized polymer (2 wt%) was added to BT-DF, and the optimal conditions were obtained according to the minimum value of FL_API_ at 200 °C. The results of the orthogonal test are shown in Table 1.

As shown in Table 1, orthogonal experiments and range analysis were used to study the effect of synthesis conditions on filtration loss. According to the range R-value, the monomer molar ratio and reaction temperature of AADN had the most significant influence on the filtration loss, while the initiator concentration and the total monomer concentration had little effect. The monomer molar ratio affects the role of functional groups in the polymer. For example, more amide and cationic groups can form better adsorption with BT particles, while more sulfonic groups can provide a better hydration effect. This can inhibit the aggregation of BT particles and facilitate the formation of the low-permeability filter cake. According to the K-value, the optimal reaction conditions can be determined as follows: n_AM_:n_AMPS_:n_DMDAAC_:n_NVCL_ = 4:6:1:2, reaction temperature 55 °C, initiator concentration 0.5%, monomer concentration 30%, pH value 7, reaction time 4 h. As shown in Table 2, the performance of AADN under the optimum synthesis conditions was better than that under other experimental conditions. Therefore, AADN was synthesized and used in experiments under the optimum conditions.

### 2.2. Characterization of AADN

**Figure 1:**
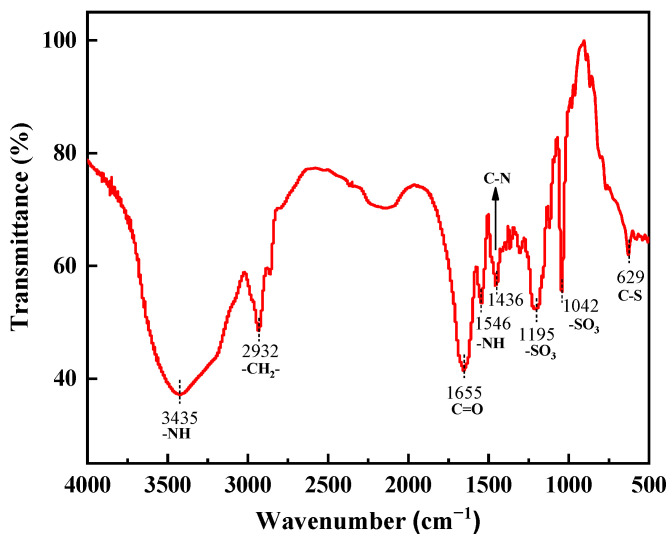
FTIR spectrum of AADN.

The molecular structure of AADN was verified by FTIR and ^1^H NMR analysis. The FTIR spectrum is shown in Figure 1. The absorption peak of the -NH stretching band in AM and AMPS is 3435 cm^−1^. An absorption peak at 2932 cm^−1^ indicates that the methylene -CH_2_- content of AADN is high. The stretching vibration peak at 1655 cm^−1^ belongs to the C=O of tertiary amide in AM, AMPS, and NVCL. The 1546 cm^−1^ peak is attributed to the deformed vibration of the amide group N-H, while the C-N bond in the five-membered heterocycle of DMDAAC has an absorption peak at 1436 cm^−1^ [29]. The absorption vibration peak at 1195 cm^−1^ and 1042 cm^−1^ are S=O stretching vibrations of the sulfonic group in AMPS, and the peak at 629 cm^−1^ conforms to the absorption vibration peak of the C-S bond on AMPS. No vibration peak of the C=C double bond at 1600–1640 cm^−1^ indicates that all reaction monomers were involved in the synthesis reaction and no monomer residue [30]. The results of AADN FTIR spectra show that the synthesized product is consistent with the designed structure.

**Figure 2:**
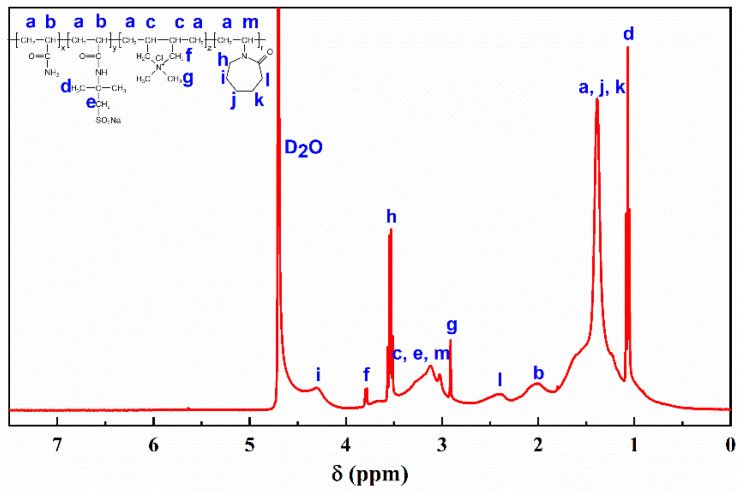
^1^H NMR spectrum of AADN.

Figure 2 shows the ^1^H NMR spectrum of AADN, and the characteristic peaks have been marked in the spectrum, where 4.709 ppm is the chemical shift of solvent D_2_O, 1.068–2.409 ppm corresponds to the chemical shifts of C-2(CH)_3_ 3 (in AMPS), -CH_2_-CH_2_-CH_2_- (in NVCL) and -CH_2_-CH- (in polymer backbone), respectively, 2.913–3.263 ppm represents the chemical shifts of N-CH_3_ (in DMDAAC) and -CH_3_-SO_3_ (in AMPS), and 3.531–4.307 ppm is designated as the chemical shift of N-CH_2_-CH_2_- (in NVCL) and -CH_2_-N^+^ (in DMDAAC), respectively. The comprehensive FTIR and NMR spectra analysis indicates that the product contained all monomer characteristic absorption peaks and chemical shifts, and AADN was the target product.

**Figure 3:**
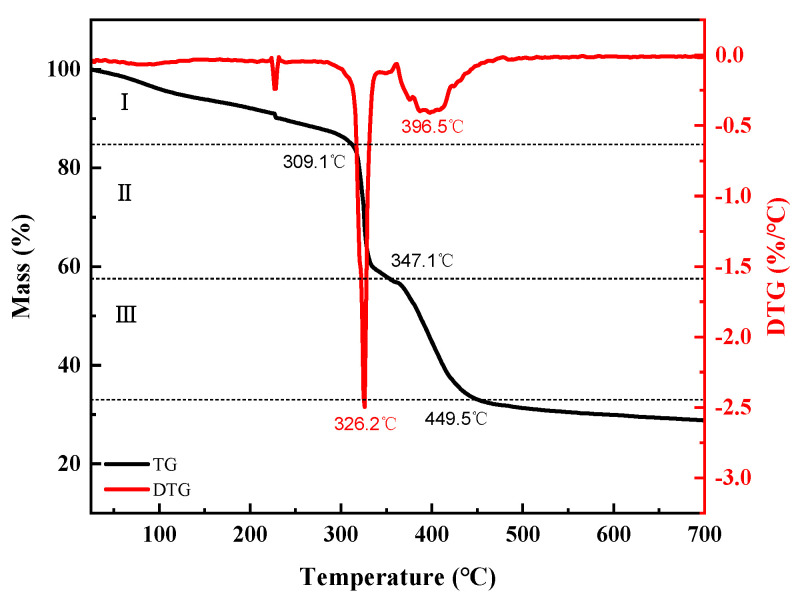
TGA curve of AADN.

The thermal stability of AADN was analyzed by TGA. As shown in Figure 3, the weight loss process can be divided into three stages from 25 °C to 700 °C. Stage I is from 25 °C to 309.1 °C. In this stage, the mass decreased slowly with the increase of temperature, mainly because the polymer AADN contains a large number of strong polar hydrophilic groups, such as amide groups and sulfonic groups, which make the polymer adsorb free water in the air. As the temperature rose, free water and volatile substances gradually volatilized, resulting in some mass loss [14]. Stage II is from 309.1 °C to 347.1 °C. The mass loss in this stage was due to the thermal decomposition of the amide and sulfonic groups in the polymer molecular chain and the break of the molecular side chain from the main chain. The maximum trough of the DTG curve occurred at 326.2 °C at this stage, indicating that the mass loss rate of AADN was greatest at this temperature. Stage III is from 347.1 °C to 449.5 °C. With a further increase in temperature, the C-C bonds of the polymer main chain began to break and decompose. The results show that the functional groups of the polymer did not decompose before 309.1 °C, indicating that the polymer has good thermal stability and is suitable for high-temperature conditions.

### 2.3. Performance Evaluation of Polymer AADN in WBDFs

#### 2.3.1. Rheological and Filtration Properties of AADN in BT-DF

**Figure 4:**
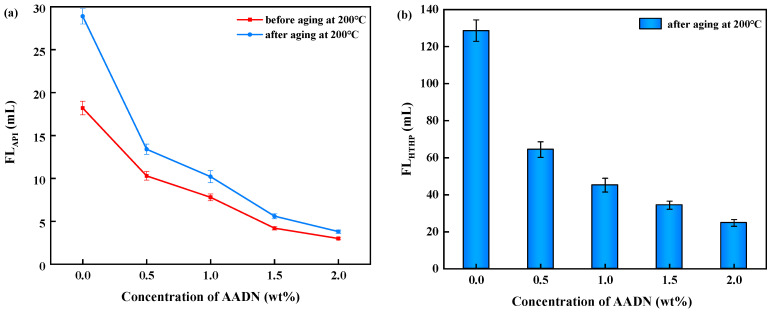
Effect of different AADN concentrations on filtration performance of BT-DF: (**a**) FLAPI before and after aging at 200 °C for 16 h; (**b**) FLHTHP before and after aging at 200 °C for 16 h.

Table 3 and Figure 4 show the rheological and filtration loss properties of BT-DF with different concentrations of AADN before and after thermal aging. It can be observed from Table 3 and Figure 4 that when the concentration of AADN increased from 0 to 2 wt%, the rheological parameters of BT-DF increased gradually while the filtration loss decreased continuously. As shown in Table 3, as the concentration of AADN increased to 2 wt%, the rheological parameters of BT-DFx, including *AV* (from 11.5 to 83.5 mPa·s), *PV* (from 7 to 45 mPa·s), *YP* (from 4.5 to 38.5 Pa) and *YP/PV* (from 0.64 to 0.86 Pa/(mPa·s)), increased significantly compared to the initial BT-DF. Due to the thermal oxidative degradation of polymers during high-temperature and oxygen aging, the interaction between polymers and BT particles was weakened. After thermal aging at 200 °C, the rheological parameters of BT-DF_2_ (*AV*: 76.5 mPa·s, *PV*: 50 mPa·s, *YP*: 26.5 Pa and *YP/PV*: 0.53 Pa/(mPa·s), respectively) decreased maintained a good effect. Especially with higher *AV* and *YP*, this shows that AADN still had excellent functions of increasing viscosity and cutting, suspending, and carrying cuttings at high temperatures [31]. This is attributed to the polymer AADN containing amide groups and cationic quaternary ammonium groups, forming a spatial network structure through hydrogen bonding and electrostatic adsorption bridging with BT particles to achieve the effect of increasing viscosity and increasing shear [32]. In addition, the ring-opening reaction of NVCL at high temperature leads to the formation of secondary amine structures on the polymer main chain, which enhances adsorption capacity through hydrogen bond formation [33]. As can be seen from Figure 4, the filtration loss of BT-DF_x_ (FL_API_ from 28.9 to 3.8 mL, FL_HTHP_ from 128.6 to 24.8 mL, respectively) decreased sharply as AADN increased to 2 wt% in BT-DF. Because the polymer AADN contains spatial ring structures such as DMDAAC [34] and NVCL [35], the thermal stability of AADN was improved. At high temperatures, the polymer is fully adsorbed on the surface of BT particles, and the sulfonic groups on the polymer AADN chain endow AADN with strong high-temperature dispersion ability [36]. This increases the thickness of the hydration film on the surface of BT particles, promoting the hydration and dispersion of BT particles, and maintaining proper particle gradation, thus forming a dense filter cake and reducing permeability.

#### 2.3.2. Salt Resistance of AADN

**Figure 5:**
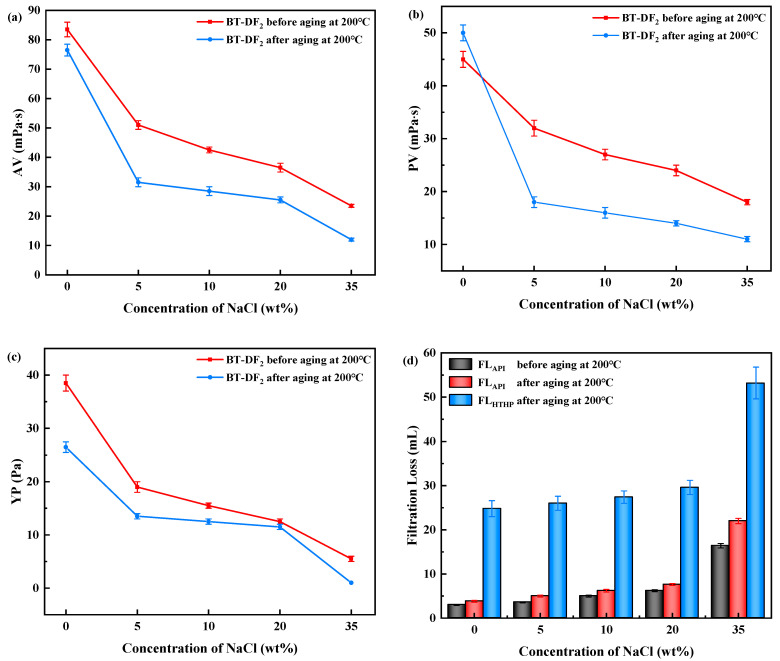
The performance of BT-DF2 under different NaCl concentrations: (**a**–**c**) Rheological parameters of BT-DF2 under different concentrations of NaCl; (**d**) Filtration loss of BT-DF2 under different con-centrations of NaCl.

High-temperature formations are usually accompanied by high-salt conditions, which can seriously affect the performance of drilling fluids. The invasion of a high-concentration NaCl would shield the electrostatic interaction between BT particles and cause flocculation of BT-DF, thereby preventing the formation of a dense filter cake. To evaluate the salt resistance of AADN, the rheological parameters and filtration loss of BT-DF_2_ at different concentrations (0–35 wt%) of NaCl were measured. The test results are shown in Figure 5. It can be seen from Figure 5a–c that as the concentration of NaCl increased, the rheological parameters *AV*, *PV*, and *YP* of BT-DF_2_ all decreased sharply at first and then decreased slowly. However, when the NaCl concentration was 20 wt%, BT-DF_2_ still had a usable effect (*AV*: 25.5 mPa·s, *PV*: 14 mPa·s, and *YP*: 11.5 Pa, respectively). This is because the intrusion of Na^+^ destroys the structure formed by BT particles to a certain extent and weakens the electrostatic repulsion between polymer groups, resulting in polymer curling up [37,38]. The intrusion of Na^+^ shields the electrostatic interaction between BT particles, making BT particles flocculate, and forms a virtual and thick filter cake. With the increased NaCl concentration, the filtration loss of BT-DF_2_ increased gradually, reaching the maximum at 35 wt% NaCl. However, with the introduction of AADN, the filtration reduction effect of BT-DF_2_ (FL_API_: 7.6 mL, FL_HTHP_: 29.6 mL, respectively) was still significant when the NaCl concentration was 20 wt%. This shows that the polymer AADN has excellent resistance to temperature and NaCl because AADN contains heterocyclic units (NVCL and DMDAAC) to maintain sufficient rigidity of the polymer and inhibit the coiling and conformational transformation of the polymer chain under the condition of high-temperature and high-salt. In addition, the sulfonic acid groups in AMPS are entirely ionized in an aqueous solution with a high ionization constant, and can chelate with metal cations [39], thus reducing the influence of salts and promoting the hydration and dispersion properties of the polymer [12]. The synergistic effect of the two ensures the rheological regulation and filtration control function of the polymer AADN in a high-temperature and high-salt environment.

### 2.4. Mechanism Analysis of Filtration Loss Control

#### 2.4.1. FTIR Analysis

**Figure 6:**
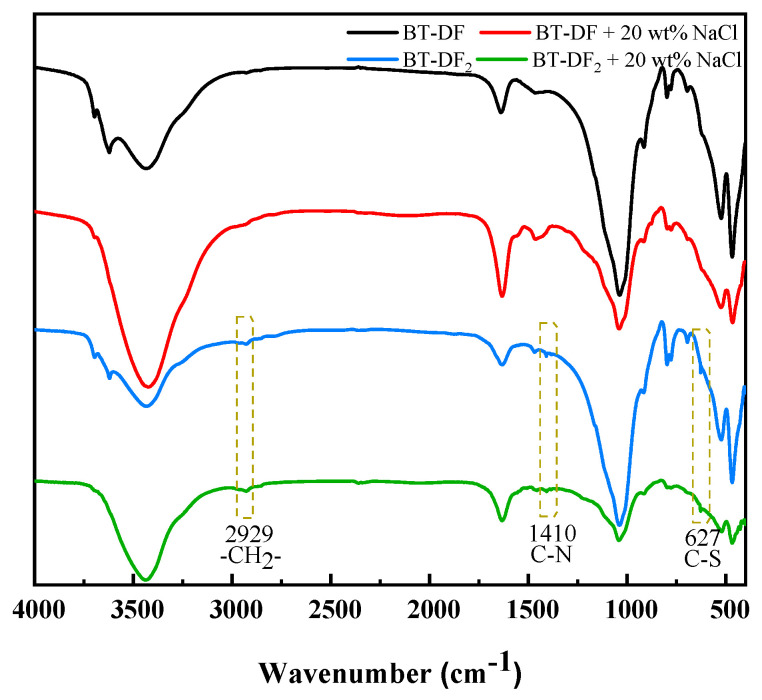
FTIR spectra of BT in different BT-DF (after aging at 200 °C for 16 h).

The adsorption between the polymer and BT is essential for the polymer to play its role [40]. Only when enough polymer molecules are adsorbed on the surface of BT particles can the filtrate of WBDF be effectively reduced. The adsorption of polymer AADN on the surface of BT after aging was explored by FTIR, and the results are shown in Figure 6. The BT-DF curve is a typical bentonite spectrum. The band at 3622 cm^−1^ is attributed to the O-H stretching vibration peak in Al-O-H. The stretching and bending vibrations of interlayer water molecules were observed at 3464 and 1640 cm^−1^, respectively. Si-O-Si antisymmetric stretching vibration occurs at 1038 cm^−1^ and Si-O bending vibration is at 468 cm^−1^ [41]. It can be observed that compared with the original BT-DF, three new characteristic peaks appeared in BT-DF_2_ containing the polymer AADN. The characteristic peak at 2929 cm^−1^ belongs to the stretching vibration of -CH_2_- in AADN. The characteristic peak at 1410 cm^−1^ is attributed to the C-N bond on the amide group. The characteristic peak at 627 cm^−1^ matches the C-S bond in the sulfonic group, which coincides with some characteristic peaks of AADN. In addition, it can be seen that the characteristic peaks of BT-DF_2_ containing 20 wt% NaCl are consistent with those of BT-DF_2_. This shows that even under high-temperature and high-salt conditions, the polymer AADN can still effectively adsorb and play a role with BT particles.

#### 2.4.2. XRD Analysis

**Figure 7:**
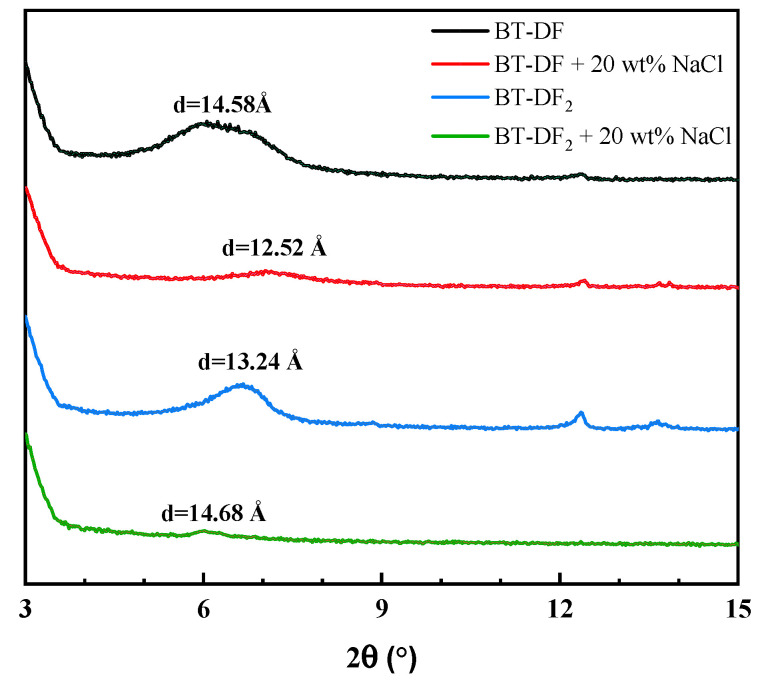
XRD of different BT-DF (after aging at 200 °C for 16 h).

The interlayer distance (d_001_) can effectively characterize the hydration dispersion effect of BT particles [42]. The interlayer distance of BT particles was analyzed by XRD, as shown in Figure 7. After aging at 200 °C, the d_001_ of BT in the original BT-DF was 14.58 Å, and that of BT in BT-DF2 with AADN was reduced to 13.24 Å. This may be due to the coating of BT particles by viscous AADN, which reduces the interlamellar distance of BT particles. With the addition of NaCl, the water activity and osmotic pressure decreased successively [43], decreasing d_001_ to 12.52 Å, which reduced the hydration effect of BT particles. With the addition of AADN, which provides BT-DF with a salt-resistance effect that counteracts the effect of NaCl on BT particles, d_001_ increased to 14.68 Å, and promoted the hydration and dispersion of BT particles. This proves that the polymer AADN has a good effect on BT-DF.

#### 2.4.3. Zeta Potential Analysis

**Figure 8:**
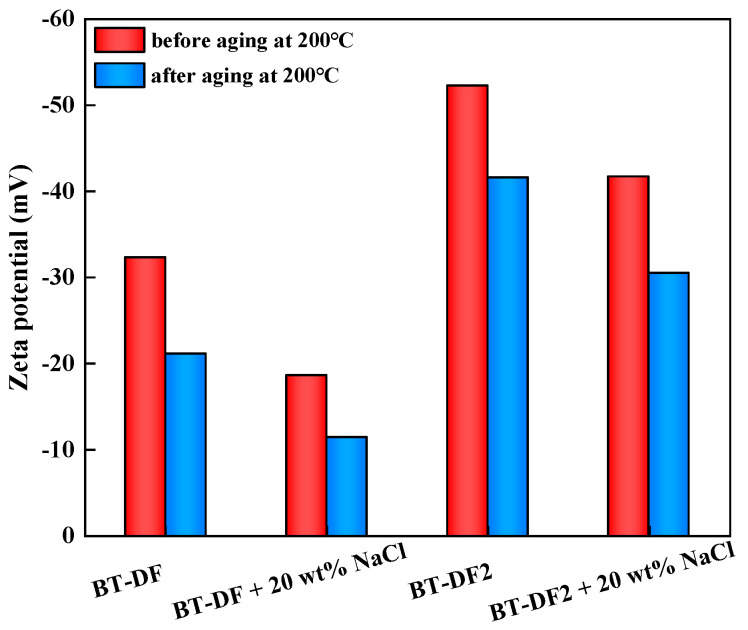
Zeta potential of different BT-DF (before and after aging at 200 °C).

WBDFs is a complex thermodynamically unstable colloidal dispersion system, and its dispersion stability is crucial. Zeta potential can be used to study the effect of polymer AADN on the dispersion stability of WBDFs. Generally, when the absolute value of the Zeta potential is higher than 30 mV, the dispersion stability of WBDFs is good [44]. The Zeta potential of BT-DF before and after aging at 200 °C was measured, and the results are shown in Figure 8. The absolute values of Zeta potentials of BT-DF and BT-DF containing 20 wt% NaCl decreased after aging, indicating that high temperature aggravated the thermal motion of water molecules, which made the hydration film of bentonite thinner, such that particles were more likely to coalesce, and the dispersion stability became worse. After adding the polymer AADN, the absolute value of the Zeta potential increases obviously. The polymer adsorbs onto BT particles through hydrogen bonding and electrostatic attraction, which thickens the diffuse double layer of BT particles, inhibits the aggregation of BT particles, maintains the proper particle gradation of BT particles, and forms a thin and dense filter cake, which significantly reduces the filtration loss. After adding NaCl, the absolute value of Zeta potential decreased due to electrostatic shielding after Na^+^ entered, and the diffuse double layer of the BT particles was compressed. The repulsive force between particles was weakened, and the structural stability of the “card house” formed by BT particles became worse. When the polymer AADN was added to the brine slurry, the absolute value of the Zeta potential increased significantly due to the reverse polyelectrolyte effect of the zwitterionic polymer [45], the polymer chain gradually extended, and the anionic groups reduced the electrostatic shielding effect generated by Na^+^, inhibiting aggregation of BT particles. The results show that AADN can maintain the dispersion and stability of WBDFs at high temperatures and in an electrolyte solution.

#### 2.4.4. Particle Size Distribution Analysis

**Figure 9:**
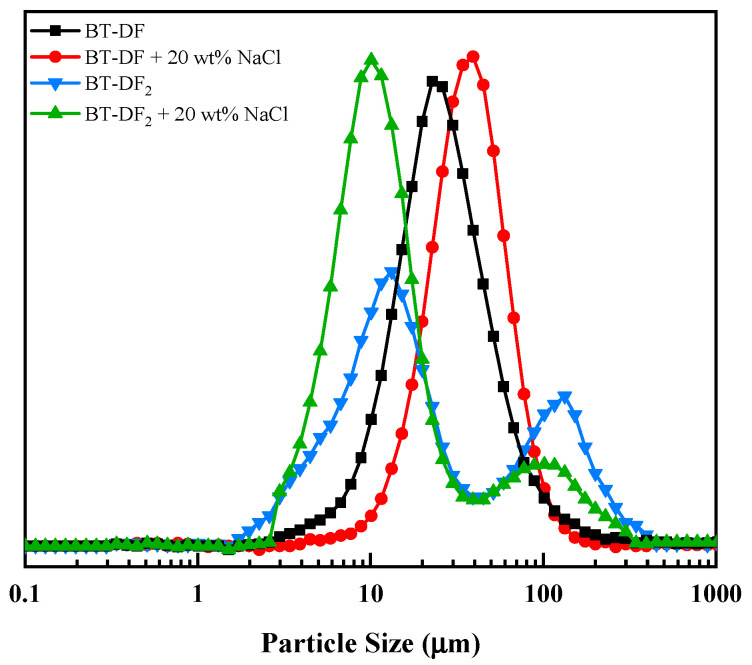
Different BT-DF particle size distributions after aging at 200 °C for 16 h.

Reasonable particle size distribution is conducive to forming a dense filter cake to improve the filtration performance of drilling fluids [46]. A drilling fluid with bimodal particle size distribution can better form a high-quality filter cake than a drilling fluid with a single peak particle size distribution. Figure 9 shows the effect of polymer AADN and cations (Na^+^) on the particle size of BT-DF after aging at 200 °C. The particle size distribution range of BT-DF and BT-DF containing 20 wt% NaCl became narrow, and D_50_ was 17.19 μm and 18.43 μm, respectively. The combined effect of high-temperature and inorganic cations promoted the flocculation and aggregation of BT particles, destroying the distribution range of BT particles, which is not conducive to forming dense filter cake, and reduces permeability. After adding the polymer AADN, the D_50_ decreased to 9.47 μm and 8.12 μm, respectively. The particle size distribution curve of the drilling fluid shifted to the left, and changed from a single peak to a double peak, and the particle size distribution range became wider. This shows that AADN has a good adsorption and dispersion promotion effect on BT particles, which can effectively prevent BT particles from agglomerating to form large particles under high-temperature and high salt to form a dense filter cake and reduce filtration volume.

#### 2.4.5. Micro-Morphology of Filter Cake

**Figure 10:**
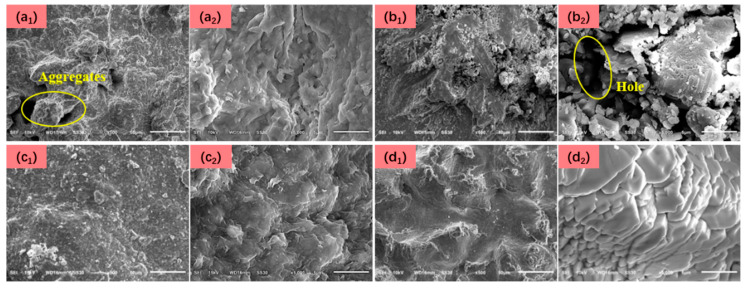
SEM image of the filter cake: (**a_1_**,**a_2_**) BT-DF; (**b_1_**,**b_2_**) BT-DF + 20 wt% NaCl; (**c_1_**,**c_2_**) BT-DF2 and (**d_1_**,**d_2_**) BT-DF2 + 20 wt% NaCl.

Using a filtration loss reducer benefits the formation of a smooth and dense filter cake in drilling fluid. The micro-morphology of the filter cake formed after aging at 200 °C was observed by SEM, as shown in Figure 10. As can be seen from Figure 10a,b, BT particles in WBDFs aggregated under the action of high temperature and NaCl, especially when NaCl invaded and caused a large number of cracks and holes in the filter cake, resulting in filtration loss (>28.9 mL) (Figure 4a). The filter cake formed after adding the polymer AADN (Figure 10c) was different. The surface of the filter cake was smooth and tightly packed, indicating that the polymer formed an effective adsorption layer on the surface of BT particles, which strengthened the hydration effect of the BT particles and maintained stable dispersion, as well as plugging delicate pores, dramatically reducing the filtration loss (3.8 mL) (Figure 4a). The filter cake maintained a similar morphology even when subjected to NaCl intrusion (Figure 10d), and there were no large-scale cracks and holes. It was verified that the polymer AADN can effectively improve the filter cake compactness and reduce filtration loss under high-temperature and high-salt conditions.

#### 2.4.6. Filtration Control Mechanism

Usually, under alkaline conditions, the surface of bentonite is negatively charged due to lattice substitution, and bentonite is dispersed in the liquid phase through electrostatic repulsion (Scheme 2①). Polar water molecules are adsorbed on bentonite particles by intermolecular attraction, forming a hydration film on the surface of bentonite, promoting the hydration and dispersion of bentonite, and forming a uniform filter cake to control filtration loss. However, high temperature aggravates the movement of bentonite particles and water molecules, and the adsorption capacity of water molecules to bentonite particles decreases, which promotes the collision and aggregation of bentonite particles. With the intrusion of cations, the diffuse double layer on the surface of bentonite is compressed, the hydration film becomes thinner, and the aggregation and flocculation of bentonite are further intensified (Scheme 2②). The resulting filter cake is virtual and thick, and the filtration loss is out of control. Zwitterionic polymer AADN contains anionic groups (sulfonic groups), cationic groups (quaternary ammonium groups), and nonionic groups (amide groups). Amide groups form hydrogen bonds with bentonite hydroxyl groups (Si-OH and Al-OH), and cationic groups form stable ionic bonds with bentonite (Scheme 2③). When the cation invades, the sulfonic groups and the cations prevent damage to the bentonite through chelation (Scheme 2④) [12]. AADN is still adsorbed on bentonite under high-temperature and high-salt to form a spatial network structure, which improves the shear dilution of BT-DF. AADN has many vigorous hydration sulfonic groups, which thicken the hydration film of bentonite particles, promote the stable dispersion of BT-DF, maintain a reasonable particle size distribution, form a dense filter cake, and seal the permeation channel [47]. At the same time, AADN increases liquid phase viscosity and synergistically reduces the filtration volume.

### 2.5. Comparison with Other Filtration Loss Reducers

**Figure 11:**
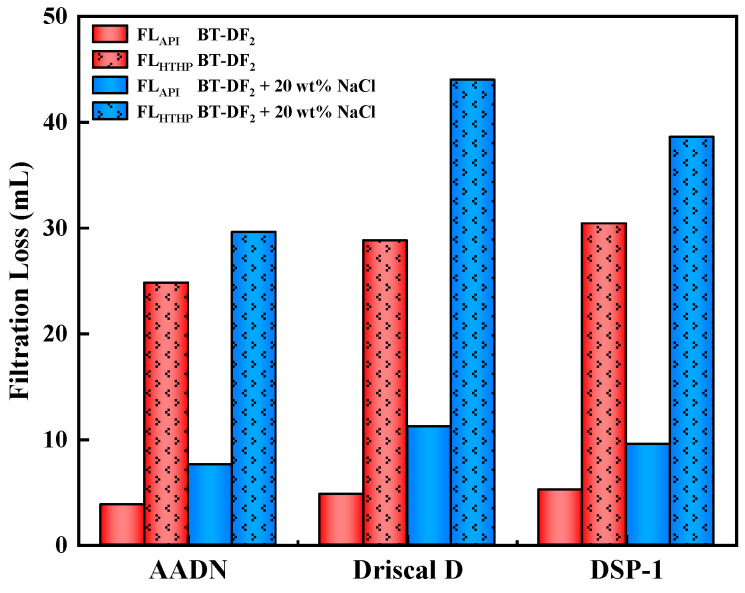
Filtration volume of different filtration loss reducers in BT-DFs (after aging at 200 °C for 16 h).

AADN (2 wt%) and commercial filtration loss reducer (2 wt% Driscal D and 2 wt% DSP-1) were added to BT-DF and containing NaCl BT-DF, respectively, and the filtration performance was evaluated. The results are shown in Figure 11. After aging at 200 °C for 16 h, the FL_HTHP_ of AADN, Driscal D, and DSP-1 in BT-DF was 24.8 mL, 28.8 mL, and 30.4 mL, respectively. Furthermore, the FL_HTHP_ in BT-DF containing NaCl was 29.6 mL, 44.0 mL, and 38.6 mL, respectively. The filtration loss control effect of AADN in two kinds of BT-DF was better than that of Driscal D and DSP-1.

## 3. Conclusions

In summary, a new type of zwitterionic polymer filtration loss reducer (AADN) with resistance to high temperature and salt pollution was synthesized by free radical polymerization of AM, AMPS, DMDAAC, and NVCL. Orthogonal experiments determined the optimal preparation conditions, and TG analysis showed that the polymer AADN has excellent thermal stability, demonstrating its potential for application in high-temperature and high-salt formations. The evaluation results of rheological and filtration performance showed that due to the reverse polyelectrolyte effect of AADN, BT-DF_2_ could resist 20 wt% NaCl pollution at 200 °C, showing excellent rheological regulation and filtration control capabilities. Compared with similar advanced products Driscal D (FL_API_ = 11.2 mL, FL_HTHP_ = 44 mL) and (FL_API_ = 9.5 mL, FL_HTHP_ = 38.6 mL), it has a better filtration control effect and better field application value. The zwitterionic polymer AADN interacts with bentonite through strong adsorption groups, and the strong hydration groups increase the diffuse double layer on the surface of bentonite particles, which promotes the stable dispersion of bentonite and confers it with excellent rheological and filtration control properties. This work provides a new strategy for applying zwitterionic polymer filtration control agents in deep high-temperature and high-salt drilling.

## 4. Materials and Methods

### 4.1. Materials

AM (99%) and NVCL (98%) were acquired by China Beijing Mreda Technology Co., Ltd., Beijing, China, AMPS (99%) and DMDAAC (60% aqueous solution) was purchased from Shanghai Macklin Biochemical Technology Co., Ltd., Shanghai, China. The initiator azobisisobutyronitrile (AIBN), anhydrous ethanol, acetone, NaOH, and NaCl were analytically pure and came from Beijing Mreda Technology Co., Ltd., China. Deionized water was self-made in the laboratory. The bentonite (BT) used to prepare drilling fluid was purchased from Hebei Huaian Bentonite Co., Ltd., Huaian, China.

### 4.2. Synthesis of Polymer AADN

Polymer AADN was synthesized by aqueous radical polymerization. A certain amount of AMPS was dissolved in deionized water, the pH value was adjusted to 7 with 20 mol/L NaOH solution, and monomers AM, DMDAAC, and NVCL were added according to a specific molar ratio and stirred until fully dissolved. Then, the mixed solution was added to the reaction vessel, N_2_ was introduced to remove oxygen, the solution stirred for 30 min after heating up to a specific temperature, and a certain mass of AIBN was added. A gel-like product was obtained after 4 h of reaction at this temperature. The product in the reaction vessel was washed with a mixed solution of anhydrous ethanol and acetone several times, precipitated, and filtered to remove unreacted monomers, dried in a vacuum at 65 °C for 24 h, and pulverized after drying to obtain the polymer AADN.

### 4.3. AADN Characterization

To explore the composition and structure of the polymer AADN, the sample was ground and mixed with potassium bromide evenly, and the mixed powder was transferred to a circular mold and pressed into a thin sheet. The infrared absorption spectrum of the polymer in the 4000~500 cm^−1^ range was measured by Fourier transform infrared spectroscopy (FTIR, Nicolet Instrument Company, Madison, WI, USA). Samples (10 mg) were fully dissolved in 0.65 mL D_2_O and then transferred to the nuclear magnetic resonance (NMR) sample tube. The proton absorption peaks of the sample were recorded by NMR (Bruker Company, Karlsruhe, Germany) to further verify the sample’s molecular structure. A thermogravimetric analyzer (TGA, NETZSCH Company, Bavaria, Germany) was used to test the thermogravimetric (TG) and derivative thermogravimetric (DTG) parameters of the sample to evaluate the thermal stability of the sample. The mass change of the sample was recorded from 25 °C to 700 °C in a nitrogen atmosphere at a heating rate of 10 °C/min.

### 4.4. Preparation and Performance Evaluation of WBDFs

#### 4.4.1. Preparation of WBDFs

To prepare the bentonite drilling fluid (4 wt% BT-DF), 400 mL tap water was added into the high-speed stirring cup, and 16 g of BT under high-speed stirring, and 0.56 g Na_2_CO_3_ were added and the solution stirred at 20 min at high speed. The stirring was stopped twice to scrape off the BT adhered to the cup wall, and the solution maintained it in an airtight container for 24 h. A certain amount of polymer filtration loss reducer was added to BT-DF (referred to as BT-DFx according to the mass percentage x) and stirred at high speed for 20 min. To test the salt-resistance performance of AADN, based on the prepared BT-DFx above, different concentrations of NaCl were added and stirred for 20 min at high speed so that AADN, BT, and NaCl were fully mixed. To study the temperature resistance of AADN, a high-temperature roller furnace (Haitongda Special Instrument Co., Ltd., Qingdao, China) was used to conduct thermal aging tests at 200 °C for 16 h. The drilling fluid properties were ultimately tested according to American Petroleum Institute (API) procedures [48].

#### 4.4.2. Performance Evaluation and Mechanism Analysis of WBDFs

Rheological properties. A six-speed rotary viscometer (Haitongda Special Instrument Co., Ltd., Qingdao, China) was used to obtain the rheological parameters of the drilling fluid. Apparent viscosity (*AV*), plastic viscosity (*PV*), and yield point (*YP*) of the drilling fluid rheological parameters were calculated by Formulas (1)–(3). In the following formula, Φ_600_ and Φ_300_ are the viscometer’s 600 and 300 rpm readings.

(1) $$AV=0.5\times \Phi_{600}\;\;\;\;\;\;\;\;\;\;\;\;\;\;\;\;\;\;\left(\mathrm{mPa}\cdot s\right)$$

(2) $$PV=\Phi_{600}-\Phi_{300}\;\;\;\;\;\;\;\;\;\;\;\;\;\;\;\;\left(\mathrm{mPa}\cdot s\right)$$

(3) $$YP=0.5\times \left(\Phi_{300}-PV\right)\;\;\;\;\;\;\left(\mathrm{Pa}\right)$$

Filtration performance. First, the API filtration loss (FL_API_) of the drilling fluid was evaluated by a drilling fluid medium-pressure filtration device (Haitongda Special Instrument Co., Ltd., Qingdao, China). The test conditions were room temperature and 100 psi pressure. Then, under the pressure difference of 500 psi, high temperature and high pressure (HTHP) filtration meter (Haitongda Special Instrument Co., Ltd., Qingdao, China) were used to measure the HTHP filtration loss (FL_HTHP_) at high temperature.

Mechanism analysis of filtration control. FTIR was used to measure the adsorption between the polymer and BT. After drying, the filter cake was ground and mixed with potassium bromide, then transferred to a circular mold and pressed into a thin sheet. The infrared absorption spectrum of the polymer in the 4000–500 cm^−1^ range was measured by FTIR. X-ray diffraction (XRD, Bruker Company, Karlsruhe, Germany) measured the interlayer distance. Filter cake crushing was carried out at a 40 kV voltage and a 20 mA current, the measurement range was 3~15°, and the scanning speed was 1°min^−1^. Then, the interlayer distance was analyzed by the Bragg equation. The Zeta potential of WBDFs was tested with a Zeta potential meter (Malvern Company, Malvern City, England). The concentration of WBDFs was about 1 g/L^−1^, and the measurements were repeated three times to get the average value. The particle size distribution of WBDFs was investigated using a laser scattering particle size analyzer (HORIBA Company, Kyoto, Japan), and the solid phase concentration was about 20 g/L^−1^. The micro-morphology of the filter cake was observed by a scanning electron microscope (SEM, JEOL, Kyoto, Japan). The filter cake was dried naturally and plated with gold before observation.

## Figures and Tables

**Scheme 2:**
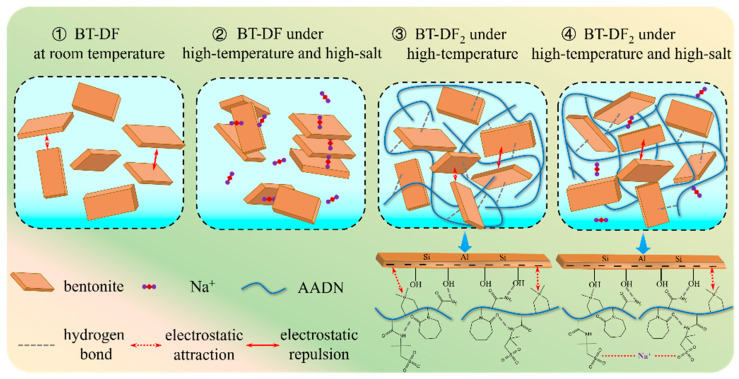
Schematic diagram of filtration loss control mechanism.

**Table 1:** Orthogonal experimental design and results of polymer AADN synthesis conditions.

Number	Mole Ratio AM: AMPS: DMDAAC: NVCL	Temperature °C	Initiator %	Monomer Concentration %	FL_API_ mL
1	8:2:1:2	45	0.3	20	4.9
2		55	0.4	25	7.5
3		65	0.5	30	6.2
4	6:4:1:2	45	0.4	30	4.5
5		55	0.5	20	5.6
6		65	0.3	25	4.0
7	4:6:1:2	45	0.5	25	6.3
8		55	0.3	30	7.0
9		65	0.4	20	5.4
K_1_	6.200	5.233	5.233	5.300	
K_2_	4.633	6.700	5.800	5.867	
K_3_	6.233	5.133	6.033	5.900	
R	1.600	1.567	0.800	0.600	

**Table 2:** Optimal synthesis conditions and performance of AADN.

Mole Ratio AM: AMPS: DMDAAC: NVCL	Temperature °C	Initiator %	Monomer Concentration %	FL_API_ mL
4: 6: 1: 2	55	0.5	30	3.8

**Table 3:** The rheological properties of BT-DF with different AADN concentrations.

Formulation	Test Conditions	*AV* mPa·s	*PV* mPa·s	*YP* Pa	*YP/PV* Pa/(mPa·s)
BT-DF	before aging at 200 °C	11.5	7	4.5	0.64
	after aging at 200 °C	8.5	5	3.5	0.70
BT-DF + 0.5 wt% AADN	before aging at 200 °C	30.5	17	13.5	0.79
	after aging at 200 °C	16.5	12	4.5	0.38
BT-DF + 1 wt% AADN	before aging at 200 °C	56.5	31	25.5	0.82
	after aging at 200 °C	35	24	11	0.46
BT-DF + 1.5 wt% AADN	before aging at 200 °C	69	37	32	0.86
	after aging at 200 °C	55	32	23	0.72
BT-DF + 2 wt% AADN	before aging at 200 °C	83.5	45	38.5	0.86
	after aging at 200 °C	76.5	50	26.5	0.53

## Data Availability

Not applicable.
